# Fertility Drugs Associated with Thyroid Cancer Risk: A Systematic Review and Meta-Analysis

**DOI:** 10.1155/2018/7191704

**Published:** 2018-05-10

**Authors:** QingAn Yu, XiaoYing Lv, KunPeng Liu, DaKun Ma, YaoHua Wu, WenJie Dai, HongChi Jiang

**Affiliations:** ^1^Department of Thyroid Surgery, The First Affiliated Hospital, Harbin Medical University, Harbin 150001, China; ^2^Department of Liver Surgery, The First Affiliated Hospital, Harbin Medical University, Harbin 150001, China; ^3^Department of Ophthalmic Clinic, The First Affiliated Hospital, Harbin Medical University, Harbin 150001, China

## Abstract

Associations have been demonstrated between fertility drugs and a variety of hormone-sensitive carcinomas. The purpose of this study was to determine the relationship between fertility drugs used in the treatment of female infertility and the risk of thyroid cancer. To investigate the clinical significance of fertility drugs used for the treatment of female infertility and the risk associated with thyroid cancer, we performed a literature search using PubMed, MEDLINE, the Cochrane Library, the Web of Science, and EBSCOHOST for comparative studies published any time prior to July 21, 2017. The studies included women who were treated for infertility with fertility drugs, such as clomiphene citrate, gonadotropins, or other unspecified fertility agents, which reported the incidence of thyroid cancer as the main outcome. Eight studies were included in the meta-analyses. Among women with infertility, there was a significant positive association between thyroid cancer risk and the use of fertility drugs (relative risk [RR] = 1.35; 95% confidence interval [CI] 1.12–1.64; *P* = 0.002). Additionally, among women with infertility, the use of clomiphene citrate was associated with an increased risk of thyroid cancer compared to women who did not use fertility drugs (RR = 1.45; 95% CI 1.12–1.88; *P* = 0.005). After pooling results, we found that the parity status of infertile women using fertility drugs was not associated with thyroid cancer risk (RR = 0.99; 95% CI 0.61–1.58, *P* = 0.95). In summary, clomiphene citrate (the most commonly used fertility drug) and other fertility drugs are associated with an increased risk of thyroid cancer.

## 1. Introduction

The incidence of thyroid cancer is increasing globally. In SEER, the trend of increasing thyroid tumor incidence rates is greater than the trends seen for any other site of tumor [1]. Mortality due to thyroid tumors is also increasing [1]. Thyroid cancer is more common in women than in men, a difference that is especially evident for differentiated thyroid cancer during the reproductive years [2]. It has been reported that the occurrence of tumors in women is related to estrogen levels and pregnancy [3–7]. Therefore, the incidence of thyroid cancer in females has attracted a lot of attention.

The incidence of infertility has also increased; an estimated 9% of couples worldwide experience some form of infertility, and 56% of these couples seek medical treatment for their infertility [8]. In recent years, there has been an increase in the development of assisted reproductive technologies and other treatments to overcome infertility, so there are now more women who use fertility drugs than in the past. The use of fertility drugs that may cause alterations in endogenous hormones and multiple ovulations has raised concerns about the long-term safety of such medications. A lot of attention has focused on whether the use of fertility-enhancing drugs can have an effect on malignancies associated with breast cancer, endometrial carcinoma, ovarian cancer, and cervical cancer [9]. Several studies have investigated the relationship between the use of fertility drugs and thyroid cancer; however, their results have been contradictory [10–19]. Therefore, we conducted a meta-analysis based on the current literature, and the results are reported in this study.

## 2. Methods

We conducted a meta-analysis of studies that investigated the association between fertility drugs and thyroid cancer risk.

### 2.1. Search Strategy

We followed the guidelines on Meta-Analysis of Observational Studies in Epidemiology for conducting systematic reviews and meta-analyses of observational studies [20]. To investigate the association between fertility drugs used in the treatment of female infertility and thyroid cancer, five main databases were searched: PubMed, MEDLINE, the Cochrane Library, the Web of Science, and EBSCOHOST. Each database was searched from the date of inception to July 21, 2017. The review was restricted to articles published in English. We used the following search keywords and Medical Subject Heading terms: ((Thyroid Neoplasms) OR (Neoplasm, Thyroid) OR (Thyroid Neoplasm) OR (Neoplasms, Thyroid) OR (Thyroid Carcinoma) OR (Carcinoma, Thyroid) OR (Carcinomas, Thyroid) OR (Thyroid Carcinomas) OR (Cancer of Thyroid) OR (Thyroid Cancers) OR (Thyroid Cancer) OR (Cancer, Thyroid) OR (Cancers, Thyroid) OR (Cancer of the Thyroid) OR (Thyroid Adenoma) OR (Adenoma, Thyroid) OR (Adenomas, Thyroid) OR (Thyroid Adenomas)) AND ((infertility) OR (ovarian stimulation) OR (ovarian hyperstimulation) OR (fertility agents) OR (fertility drugs) OR (clomiphene) OR (gonadotropins) OR (gonadotropin-releasing hormones) OR (induction of ovulation) OR (in vitro fertilization) OR (assisted reproductive technology)).

### 2.2. Inclusion Criteria

The included studies had to conform to the following criteria: (1) the study consisted of females who had received an explicit and reproducible diagnosis of infertility and had been treated with fertility-enhancing drugs and who were compared with infertile untreated controls; (2) the study evaluated the association between fertility drugs and the risk of thyroid cancer; (3) the design was a cohort or case-control study; and (4) the report presented odds ratios (ORs), relative risks (RRs), or hazard ratios (HRs) with 95% confidence intervals (95% CIs). Animal research, case reports, duplicated studies, and studies not associated with fertility drugs and thyroid cancer risk were all excluded. We extracted information on study design, reporting time, sample size, type of fertility drugs used and number of treatment cycles, parity status, thyroid cancer incidence, and follow-up time from these studies.

### 2.3. Data Extraction and Quality Assessment

Two investigators independently extracted data from each included study. The data included the name of the first author, year of publication, study design, country of origins, inclusion and exclusion criteria, total sample size, number of thyroid cancer patients in the intervention and control groups, matching or adjusted factors, and RRs or HRs or ORs with 95% CIs for the estimation of thyroid cancer risk related to the use of fertility drugs. We evaluated and filtered all the included studies, grading each study according to the Newcastle–Ottawa Scale (NOS) [21]. This scale was used to assign a maximum of nine points for each study. Studies with scores of seven or greater were categorized as high-quality studies, and those with score of 6 or lower were categorized as low-quality studies. Disagreements were solved by discussions between members of the study team.

### 2.4. Statistical Analysis

We classified the selected studies, extracted data, and performed the meta-analysis using RevMan 5.3 software, which was provided by the Cochrane Library. The role of fertility drugs in thyroid cancer risk were assessed by calculating pooled RRs and 95% CIs. The between-study heterogeneity was estimated using Cochran's *Q* and *I*^2^ tests, and *P* < 0.1 and *I*^2^ > 50% implicated obvious between-study heterogeneity.

## 3. Results

### 3.1. Literature Search and Study Characteristics

**Figure 1:**
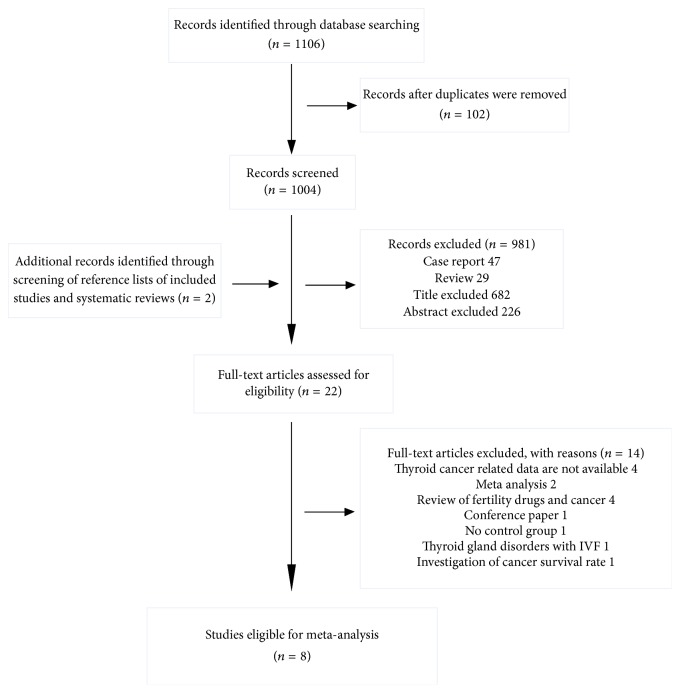
Flowchart of the study selection process.

The detailed steps regarding the search are shown in Figure 1. Overall, 1,106 records were identified, and 102 duplicate records were excluded. Two additional records were identified by screening the reference lists of included studies and systematic reviews; we excluded an additional 981 records that were case reports, review articles, research protocol articles, articles based on guidelines, the wrong type of studies, or the wrong population group. Of the 22 studies selected for potential analysis, a further 14 were excluded because four had no specific data about thyroid cancer, two were meta-analyses, four were review articles, one was a conference paper, one had no control groups, one focused on the association between thyroid gland disorders and in vitro fertilization (IVF), and one was an investigation of the cancer survival rate. Finally, eight studies (six retrospective cohort studies, one cohort study, and one case-cohort study) were included in the analysis (Table 1) [9–13, 16–18]. The studies included were Israeli (*n* = 2), Norwegian (*n* = 2), American (*n* = 2), Danish (*n* = 1), and Finnish (*n* = 1). The studies enrolled 2,215,467 patients and had a median follow-up of 16.5 years (range 7.3–30 years). The details regarding NOS for all the included studies are shown in Table 2; all the studies were categorized as high-quality studies.

### 3.2. Association between Fertility Drugs and Thyroid Cancer Risk

**Figure 2:**
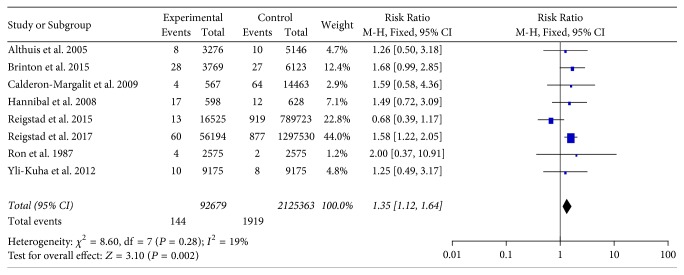
Forest plots of Risk Ratio with corresponding 95% CIs for the correlation between fertility drugs and thyroid cancer risk. CI: confidence interval.

After pooling the results of the included studies, there was a significant positive association between the risk of thyroid cancer for women who used fertility drugs versus women who did not use fertility drugs (RR = 1.35; 95% CI 1.12–1.64; *P* = 0.002), with no considerable heterogeneity (*P* = 0.28; *I*^2^ = 19%; Figure 2).

**Figure 3:**
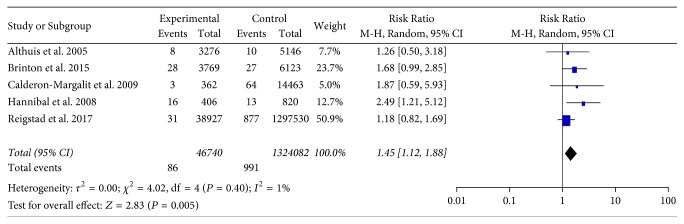
Forest plots of Risk Ratio with corresponding 95% CIs for the correlation between clomiphene citrate and thyroid cancer risk. CI: confidence interval.

As clomiphene citrate is the most widely used fertility drugs, we pooled results from five studies which described the risk of thyroid cancer after using clomiphene citrate [11–13, 18, 19]. Among women with infertility, the risk of thyroid cancer was significantly greater for those who used clomiphene citrate versus women who did not use fertility drugs (RR = 1.45; 95% CI 1.12–1.88; *P* = 0.005), with no considerable heterogeneity (*P* = 0.40; *I*^2^ = 1%; Figure 3).

### 3.3. Parity Status

**Figure 4:**
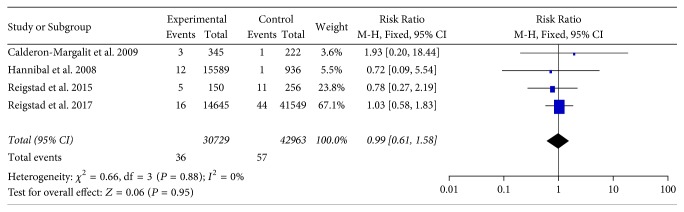
Forest plots of Risk Ratio with corresponding 95% CIs for the correlation between parity status of infertile women who were treated with fertility drugs and thyroid cancer risk. CI: confidence interval.

Hannibal et al. [12] showed that parous versus nulliparous women who used fertility drugs had a higher risk of thyroid cancer (RR = 3.09; 95% CI 1.21–7.88). Therefore, we stratified the results based on parity status. In the meta-analysis, we observed that there was no association between the use of fertility drugs and thyroid cancer risk in parous versus nulliparous women (RR = 0.99; 95% CI 0.61–1.58; *P* = 0.95), with no considerable heterogeneity (*P* = 0.88; *I*^2^ = 0%; Figure 4).

## 4. Discussion

This study shows that the use of fertility drugs by infertile women increases their risk of developing thyroid cancer.

The number of infertile women who receive treatment for infertility is increasing, as the incidence of infertility increases. Some studies have suggested that there are relationships between the use of fertility therapy and the risk of cancer, especially for breast cancer, endometrial cancer, and other hormone-related carcinomas [19, 22, 23]. Thyroid cancer is also a hormone-related cancer; elevated estrogen levels can promote the growth of differentiated thyroid cancer [24–28], which may be related to the estrogen receptor pathway [29, 30]. It is unknown whether the use of fertility drugs by infertile women is associated with an increased risk of thyroid cancer. Therefore, we reviewed all related published studies for this meta-analysis. Through database retrieval and screening, we enrolled all eligible studies, which included eight cohort studies and involved a total of 2,215,467 infertile patients, of whom 92,679 received fertility treatment. A pooled analysis showed that fertility therapy increased the risk of thyroid cancer; these results were statistically significant with no heterogeneity (RR = 1.35; 95% CI 1.12–1.64; *P* = 0.002).

Of the eight cohort studies, seven identified the fertility treatment and medication. One of the interventions was IVF [14], but the associated report did not explicitly detail what fertility drugs were used. Another study used IVF, intracytoplasmic sperm injection (ICSI), and other treatment modalities but also did not explicitly state what kinds of fertility drugs were used [17]. We carried out a further analysis on the remaining five studies, in which the main fertility drugs used were clomiphene citrate, gonadotropins, progesterone, human chorionic menopausal (hCG), and gonadotropin-releasing hormone (GnRH). Clomiphene citrate is the most widely used fertility drug. Thus, we carried out further analyses to investigate the impact of clomiphene citrate for the treatment of infertility. Among the study participants with infertility, we found that those who used clomiphene citrate had a higher risk of thyroid cancer than those who did not (RR = 1.45; 95% CI 1.12–1.88; *P* = 0.005). Due to lack of specific statistical data, we were unable to assess the individual associations of gonadotropins, progesterone, hCG, and GnRH with thyroid cancer risk. Hannibal et al. [12] showed that progesterone is associated with thyroid cancer (RR = 10.14; 95% CI 1.93–53.34); however, Brinton et al. [18] and Althuis et al. [11] showed that progesterone is not associated with an increased thyroid cancer risk. Additionally, Hannibal et al. [12] showed that gonadotropins, hCG, and GnRH are not associated with an increased thyroid cancer risk.

Parity status is an important adjustment factor when studying fertility treatment. Althuis et al. [11], Brinton et al. [18], and Reigstad et al. [19] showed that nulliparous women who used fertility drugs had a higher risk of thyroid cancer when compared with parous women. On the other hand, Hannibal et al. [12] arrived at the opposite finding. After pooling the results, we found that the parity status of infertile women using fertility drugs was not associated with thyroid cancer risk (RR = 0.99; 95% CI 0.61–1.58, *P* = 0.95).

This study had several limitations. Relatively few cohort studies have been published regarding the use of fertility drugs and the risk of thyroid cancer; more cohort studies are needed to further confirm the association between the use of fertility drugs and the risk of developing thyroid cancer. Additionally, because of the relatively small quantity of available data, there was no adjustment for confounding factors related to infertility. Finally, we did not perform a detailed investigation of the doses and cycles of the administered fertility drugs. Althuis et al. [11] and Brinton et al. [18] showed that the doses and cycles of fertility drugs use were not associated with thyroid cancer risk. And Hannibal et al. [12] found for all groups of fertility drugs there were no substantial differences in thyroid cancer risk according to number of cycles of use.

## 5. Conclusion

Increased thyroid cancer risk is associated with the use of fertility drugs, including the most commonly used fertility drug, clomiphene citrate. The parity status of infertile women who use fertility drugs is not related to their risk of developing thyroid cancer. There is a need for further epidemiological studies with large sample sizes to confirm the magnitude of the association between the use of fertility drugs and the risk of developing thyroid cancer.

## Figures and Tables

**Table 1:** Characteristics of all included studies.

Study	Location	Study size	Study design	Enrollment period	Fertility treatment	Matched/adjusted factors	Follow-up (years)
Ron et al. [10]	Israel	2,575	Retrospective cohort study	1964–1974	Unknown	Unknown	12.3

Althuis et al. [11]	USA	8,422	Retrospective cohort study	1965–1988	Clomiphene, gonadotropins	Study site, age at follow-up, calendar year of follow-up, gravidity at entry, parity at follow-up	18.8

Hannibal et al. [12]	Denmark	54,362	Case-cohort study	1963–1998	Clomiphene, gonadotropins, progesteronr, hCG, GnRH	Age at first live birth, parity status	8.8

Calderon-Margalit et al. [13]	Israel	15,030	Retrospective cohort study	1974–1976	Clomiphene, gonadotropins, other unknown fertility drugs	Age at first birth, geographic origin, social class, education, parity, time to conception, mean body mass index	29

Yli-Kuha et al. [14]	Finland	18,350	cohort study	1996–1998	IVF	Age, marital status, socioeconomic position	7.8

Reigstad et al. [17]	Norway	806,248	Retrospective cohort study	1984–2010	IVF, ICSI, others	Age at start follow-up, parity at entry, method of ART, duration of follow-up	7.3

Brinton et al. [18]	USA	9,892	Retrospective cohort study	1965–1988	Clomiphene, gonadotropins, combination	Race, reproductive status at first clinic visit, reproductive status at follow-up, number of births at follow-up, age at first birth, age at menarche, BMI at first clinic visit, ever smoke, cause of infertility	30

Reigstad et al. [19]	Norway	1,353,724	Retrospective cohort study	1960–1996	Clomiphene citrate, GnRH, gonadotropins, hCG	Birth year, parity, CC exposure, region of present residence, number of children at entry, age at start of follow-up, age at first cancer, types of treatment, number of cycles of ART, dose of clomiphene citrate	18

hCG: human chorionic gonadotropin, GnRH: gonadotropin-releasing hormone, IVF: in vitro fertilization, and ICSI: intracytoplasmic sperm injection.

**Table 2:** Newcastle–Ottawa quality assessment of cohort studies.

Study	Cohort representative	Selection of nonexposed cohort	Ascertainment of exposure	Outcome negative at start	Comparable cases and controls	Outcome assessment	Duration of follow-up	Adequate follow-up	Score
Ron et al. [10]	A		A	A	A	A	A	A	7
Althuis et al. [11]	A	A	A	A	A	A	A	A	8
Hannibal et al. [12]	A	A	A	A	A	A	A	A	8
Calderon-Margalit et al. [13]	A	A	A	A	A	A	A	A	8
Yli-Kuha et al. [14]	A	A	A	A	B	A	A	A	9
Reigstad et al. [17]	A	A	A	A	A	A	A	A	8
Brinton et al. [18]	A	A	A	A	A	A	A	A	8
Reigstad et al. [19]	A	A	A	A	B	A	A	A	9
